# Factors associated with COVID-19 in-hospital death and COVID-19 vaccine effectiveness against COVID-19 hospitalization in the Philippines during pre-omicron and omicron period: A case-control study (MOTIVATE-P study)

**DOI:** 10.1017/S0950268824001845

**Published:** 2024-12-20

**Authors:** Takeshi Arashiro, Rontgene Solante, Ana Ria Sayo, Reby Marie Garcia, Marie Kris, Shuichi Suzuki, Greco Mark Malijan, Mary Jane Salazar, Mary Ann Salazar, Abby Ortal-Cruz, Grace Devota Go, Edna Miranda, Michelle Carandang-Cuvin, Joy Potenciano Calayo, Jinho Shin, Martin Hibberd, Koya Ariyoshi, Chris Smith

**Affiliations:** 1 Faculty of Infectious and Tropical Diseases, London School of Hygiene and Tropical Medicine, London, UK; 2 School of Tropical Medicine and Global Health, Nagasaki University, Nagasaki, Japan; 3 Center for Surveillance, Immunization, and Epidemiologic Research, National Institute of Infectious Diseases, Tokyo, Japan; 4 Department of Pathology, National Institute of Infectious Diseases, Tokyo, Japan; 5 World Health Organization Regional Office for the Western Pacific, Manila, Philippines; 6 Adult Infectious Diseases and Tropical Medicine Unit, San Lazaro Hospital, Manila, Philippines; 7 Epidemiology Department, San Lazaro Hospital, Manila, Philippines; 8 San Lazaro Hospital-Nagasaki University Collaborative Research Office and Laboratory, San Lazaro Hospital, Manila, Philippines; 9 Pediatrics Department, San Lazaro Hospital, Manila, Philippines; 10 Department of Laboratory, San Lazaro Hospital, Manila, Philippines

**Keywords:** severe acute respiratory syndrome coronavirus 2 (SARS-CoV-2), coronavirus disease (COVID-19), epidemiological study, vaccine effectiveness, SARS-CoV-2 variants, Philippines

## Abstract

COVID-19 vaccine effectiveness (VE) studies are limited in low- and middle-income countries. A case-control study was conducted among COVID-19 and other pneumonia patients admitted to a hospital in the Philippines during the pre-Omicron and Omicron periods. To elucidate factors associated with in-hospital death, 1782 COVID-19 patients were assessed. To estimate absolute VE for various severe outcomes, 1059 patients were assessed (869 [82.1%] COVID-19 cases; 190 [17.9%] controls). Factors associated with in-hospital death included older age, tuberculosis (adjusted odds ratio [aOR] 2.45 [95% confidence interval {95% CI} 1.69–3.57]), HIV (aOR 3.30 [95% CI 2.03–5.37]), and current smokers (aOR 2.65 [95% CI 1.72–4.10]). Pre-Omicron, the primary series provided high protection within a median of 2 months (hospitalization: 85.4% [95% CI 35.9–96.7%]; oxygen requirement: 91.0% [95% CI 49.4–98.4%]; invasive mechanical ventilation (IMV): 97.0% [95% CI 65.7–99.7%]; death: 96.5% [95% CI 67.1–99.6%]). During Omicron, the primary series provided moderate-high protection within a median of 6–9 months (hospitalization: 70.2% [95% CI 27.0–87.8%]; oxygen requirement: 71.4% [95% CI 29.3–88.4%]; IMV: 72.7% [95% CI −11.6–93.3%]; death: 58.9% [95% CI −82.8–90.8%]). Primary series VE against severe COVID-19 outcomes was consistently high for both pre-Omicron and Omicron in a setting where approximately half of the vaccinees received inactivated vaccines.

## Introduction

Coronavirus disease (COVID-19), caused by severe acute respiratory syndrome coronavirus 2 (SARS-CoV-2), has resulted in substantial morbidity and mortality globally [1]. Once the COVID-19 vaccines were rolled out based on trial results [2–7], there was a need to monitor the real-world effectiveness of the vaccines (vaccine effectiveness; VE), given concerns due to waning immunity and the emergence of variants with immune escape capacity [8–12]. There have been numerous studies to evaluate VE, mostly from high-income countries (HICs), but the evidence is very limited in low- and middle-income countries (LMICs). This is especially true for Southeast Asia (specifically, the Western Pacific Region) and Africa [13]. It was considered valuable for more LMICs, especially low- and lower-middle-income countries, to conduct VE studies for several reasons, including: (1) vaccines rolled out in LMICs differed from HICs; (2) cold chain breach may be more likely in LMICs (e.g. some vaccines required ultra-cold temperatures); (3) cumulative infection burdens were considered much higher in LMICs and this may affect VE estimates (e.g. individuals with prior infection are protected against subsequent infection/disease); (4) substantial variation in public health and social measures among countries, which may also affect VE estimates; (5) VE results in local or regional contexts may results in further vaccine confidence within and among surrounding countries; and (6) capacity building to conduct operational research to inform public health response for COVID-19 as well as future epidemics and pandemics. Also, specifically for inactivated vaccines, which were widely rolled out in LMICs, VEs against hospitalization outcomes from previous reports were highly varied, and data against the Omicron variant is especially limited [13, 14]. This variability in hospitalization outcomes may be due to different criteria for hospitalization and incidental diagnosis of SARS-CoV-2 infection during routine admission screening [15, 16]. This could potentially result in lower VE estimates against severe disease, as VE against infection is generally lower than VE against severe disease [13, 15, 16]. Therefore, we conducted a study to elucidate factors associated with in-hospital death among SARS-CoV-2-positive hospitalized patients and to evaluate COVID-19 VE against hospitalization in the Philippines during the pre-Omicron and Omicron periods. For VE estimates, we used various outcomes, including more severe and specific outcomes such as oxygen use and invasive mechanical ventilation use.

## Methods

### Study design and setting

Our study, ‘Moderate-to-severe diseases requiring Oxygen Therapy, Intubation, and Ventilation And The Effectiveness of COVID-19 vaccines in the Philippines’ (MOTIVATE-P study), is a single-centre study at San Lazaro Hospital (SLH) in Manila with two objectives: (1) to elucidate factors associated with in-hospital death among SARS-CoV-2-positive hospitalized patients; and (2) to estimate the real-world effectiveness of COVID-19 vaccines against severe disease. Outside the context of the COVID-19 pandemic, SLH is a government-retained specialty referral hospital for infectious diseases. During the COVID-19 pandemic, SLH routinely admitted patients with COVID-19 and pneumonia caused by other pathogens and routinely tested individuals admitted using polymerase chain reaction (PCR) for clinical diagnostic and screening purposes [17]. It has also been functioning as one of the main COVID-19 response sites in the country. We followed the same design as a study conducted and published previously by some of the authors in Japan [15].

### Study period

**Figure 1.**
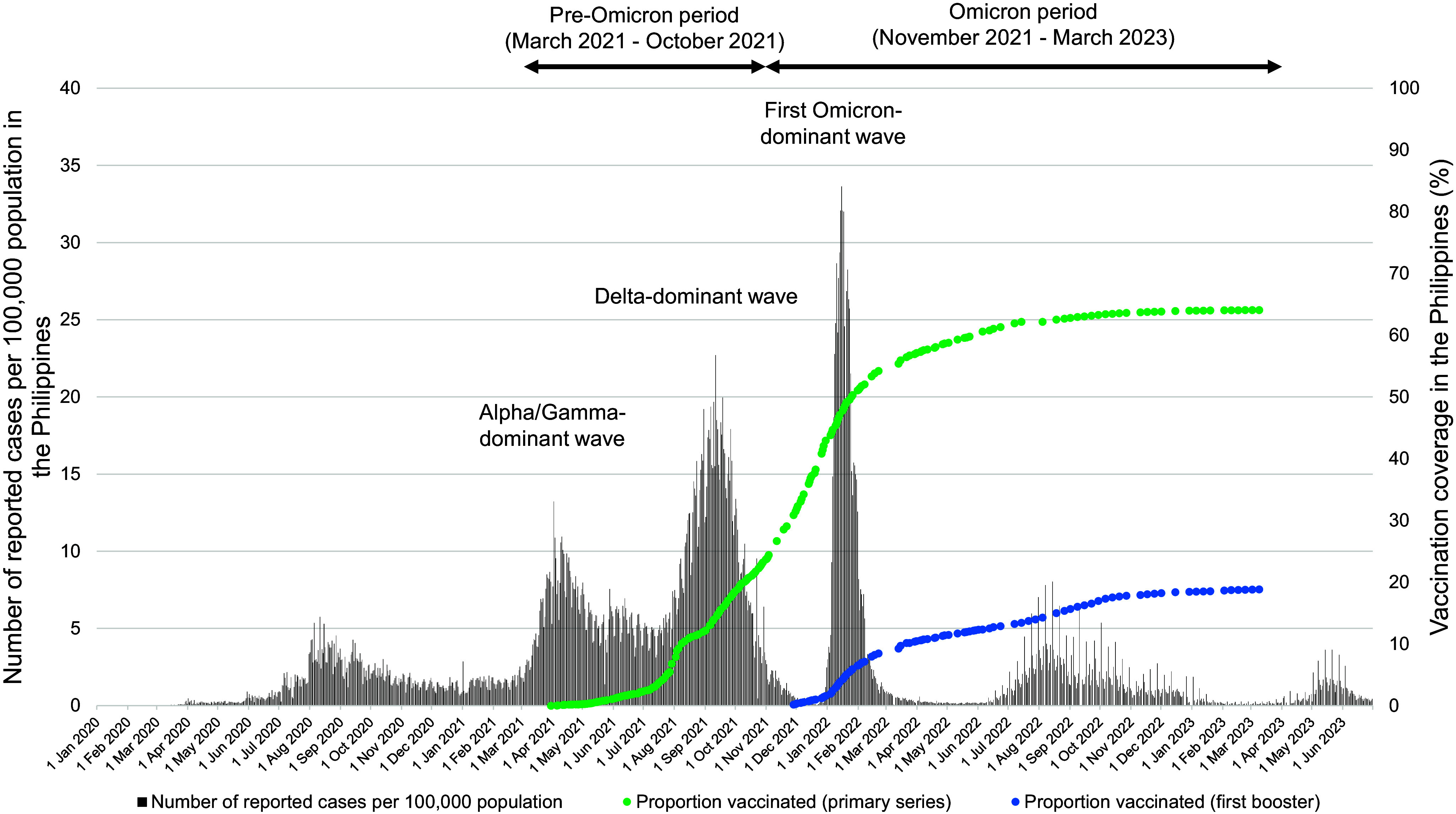
Epidemic curves of the number of reported COVID-19 cases and vaccine rollout in the Philippines. The data are likely underestimated due to reporting constraints, testing/reporting intensity varied substantially over time, and COVID-19 vaccination data are up to 9 March 2023. Source: Our World in Data (https://ourworldindata.org).

The study period was between 1 March 2021 (when the COVID-19 vaccination rollout started in the Philippines) and 31 March 2023 (before Omicron subvariant XBB became dominant). Based on genomic surveillance data, the Omicron variant was first detected in the Philippines in November 2021 and quickly replaced the Delta variant (Figure 1) [18]. Therefore, we defined 1 March to 31 October 2021 as the pre-Omicron (Alpha, Gamma, and Delta) period and 1 November 2021 to 31 March 2023 as the Omicron period. In the Philippines, the primary series (one dose for Janssen and two doses for all other vaccine types) rollout started on 1 March 2021 [19]. The primary series followed manufacturer-recommended intervals. The first booster dose rollout began on 16 November 2021 for healthcare workers (HCWs), on 22 November 2021 for senior citizens and immunocompromised persons, and on 3 December 2021 for all adults aged 18 years or above. The second booster dose rollout started on 25 April 2022 for HCWs and individuals who were ≥60 years old and on 27 July 2022 for individuals who were ≥50 years old and individuals aged 18–49 years with comorbidities.

### Inclusion and exclusion criteria

The inclusion criteria were SARS-CoV-2-positive hospitalized patients and SARS-CoV-2-negative hospitalized pneumonia patients. Pneumonia caused by tuberculosis was not included as the clinical presentation would be different from the one caused by COVID-19 pneumonia or common bacterial pneumonia with acute onset. Patients were excluded for the following reasons: symptom onset during hospitalization; tested ≥15 days before or ≥15 days after admission; and unknown test date.

### Data collection

Data, including outcomes, were collected via a review of medical charts and other relevant hospital documents by trained research nurses. Vaccination status (number of doses, vaccine type [e.g. manufacturer], and vaccination dates) was recorded from the medical charts, case investigation form (CIF), and/or other relevant hospital documents and checked for plausibility. The CIF was a form that was required to be completed when conducting SARS-CoV-2 testing during the study period and was generally filled out by referencing the vaccination card. To ensure the quality of data entry, ten charts were randomly selected soon after the initiation of the study, entered by two different nurses, and checked for consistency.

### Data description and analysis of factors associated with in-hospital death among SARS-CoV-2-positive hospitalized patients

Characteristics of SARS-CoV-2-positive hospitalized patients admitted during the study period were described overall and by pre-Omicron and Omicron period. Logistic regression was used to estimate the factors associated with in-hospital death. The model was adjusted for age group (categorical), sex, risk score categories (0, 1, 2, 3–4, 5+; categorical [detailed later]), calendar week of hospitalization (biweekly), and vaccine doses (except for the factor of interest). The risk score for severe disease developed in a study published by some of the authors in Japan was incorporated as a covariate [15, 20, 21]. Here, we assigned 2 points for the presence of either diabetes mellitus, chronic kidney disease (CKD), dementia, Down syndrome, or obesity and assigned 1 point for the presence of cardiovascular disease (including hypertension), dyslipidaemia, chronic liver disease, chronic obstructive pulmonary disease, cancer, depression/schizophrenia, stroke, tuberculosis, immunocompromised condition (HIV infection or other immunodeficiencies, or immunosuppressant use), pregnancy while hospitalized, or overweight; the points were added up to calculate the risk score for each patient.

### Additional exclusion criteria for VE analysis

For the VE analysis, patients were further excluded for the following reasons: being <50 years of age, past SARS-CoV-2 infection (based on medical chart review), and (for controls) diagnosis of pneumococcal pneumonia or influenza. The rationale for including patients who were tested up to 14 days before admission and excluding those who were tested ≥15 days before admission is that it takes from a few days to 2 weeks from symptom onset for patients to develop severe disease, and these patients may be tested right after onset and later hospitalized. The rationale for restricting to individuals ≥50 years of age was to aim for better internal validity among those most at risk of severe COVID-19, and because individuals aged 50 years and above were eligible for the second booster. This, we considered, would allow us to reduce confounding through different socioeconomic factors and vaccine prioritization. Finally, co-circulation of influenza and COVID-19 can result in biased VE estimates as the propensity to get vaccinated may be similar for COVID-19 and influenza vaccines [22]. In theory, the same concern applies to *Streptococcus pneumoniae* pneumonia and pneumococcal vaccination. Therefore, we excluded patients with pneumococcal pneumonia or influenza.

### Estimation of vaccine effectiveness

Patients who tested positive before or after admission based on the above inclusion and exclusion criteria were defined as cases; other pneumonia patients who tested negative before or after admission based on the above criteria were defined as controls.

To measure absolute VE compared to the unvaccinated, we analyzed various severe outcomes. Outcomes included all COVID-19 hospitalizations, cases requiring oxygen therapy, cases requiring invasive mechanical ventilation, death, outcomes restricting to ‘true’ severe COVID-19 (where oxygen requirement is due to COVID-19 rather than other differential diagnoses), and progression from oxygen use to mechanical ventilation or death. A ‘true’ severe COVID-19 outcome was based on the judgment of the treating physicians (chart record) and trained nurses responsible for chart review. The chart review was conducted between June 2023 and May 2024 to ensure that at least 6 months had passed since participants were hospitalized to allow for sufficient time to reach the final discharge outcome for participants.

Patient characteristics for the VE analysis dataset were described first overall then by case/control status. Vaccination status was classified by dose and/or time since vaccination.

Logistic regression was used to estimate the odds of being vaccinated among cases relative to controls. The model was adjusted for age group (categorical), sex, risk score categories (0, 1, 2, 3–4, 5+; categorical), smoking history, and calendar week of hospitalization (biweekly). These potential confounders were determined *a priori* based on published reports [10, 15]. VE was estimated using the following equation: VE = (1 – adjusted odds ratio [aOR]) × 100%. Data analyses were performed using STATA version 18.0.

### Ethics statement

Ethics approval was obtained from the San Lazaro Hospital Research Ethics Committee. Informed consent was deemed unnecessary due to the retrospective nature of the study.

## Results

### Study participants

**Figure 2.**
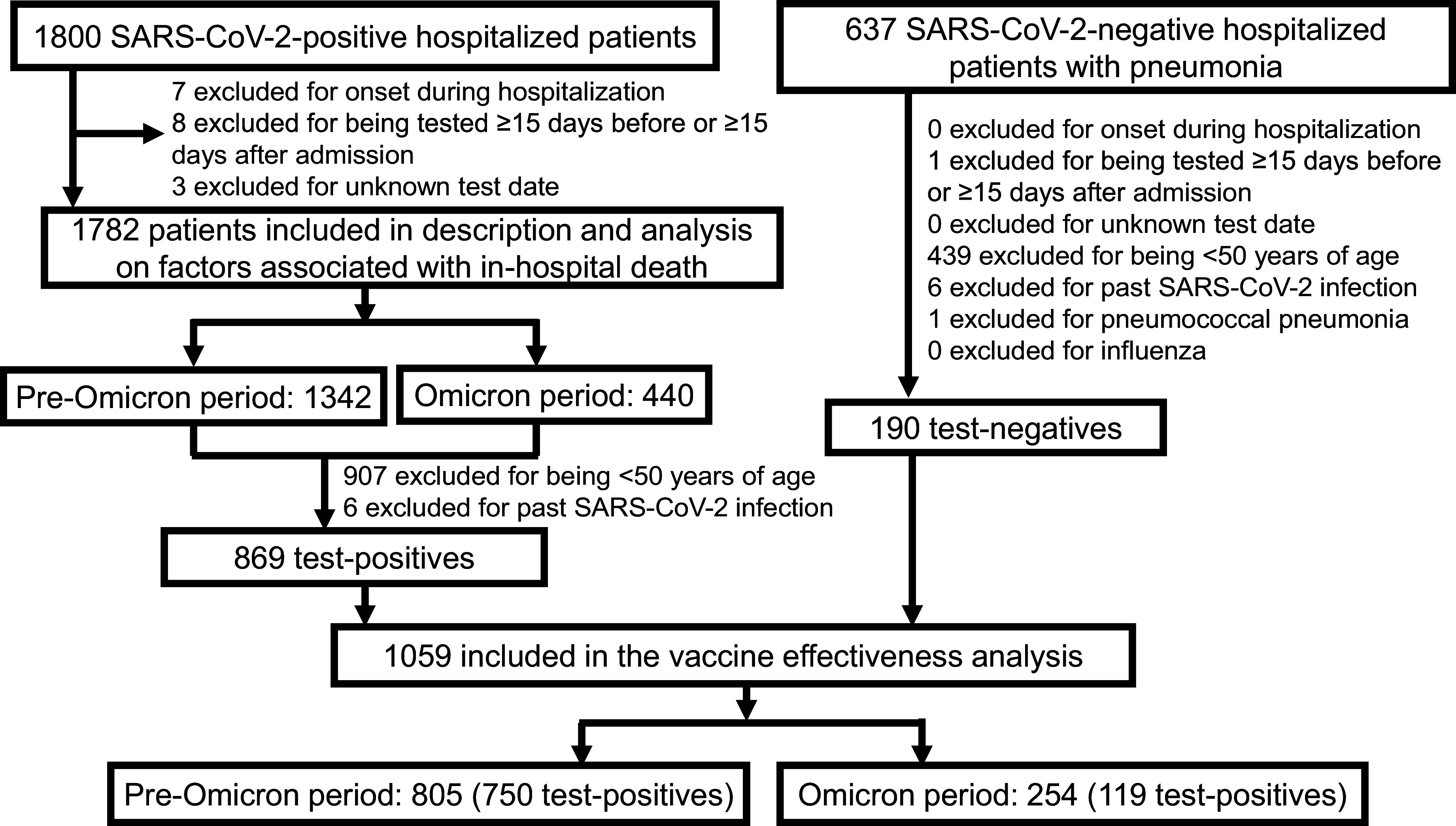
Flow diagram of the study participants.

A total of 1800 SARS-CoV-2-positive hospitalized patients and 637 SARS-CoV-2-negative hospitalized pneumonia patients were initially included. For the description of SARS-CoV-2 hospitalization, after excluding 18 patients based on exclusion criteria, the final analysis included 1782 patients: 1342 for the pre-Omicron period and 440 for the Omicron period (Figure 2). For the cases in VE analysis, after further excluding 913 patients based on exclusion criteria, the final analysis included 869 patients: 750 for the pre-Omicron period and 119 for the Omicron period. For the controls in VE analysis, after excluding 447 patients based on exclusion criteria, the final analysis included 190 patients: 55 for the pre-Omicron period and 135 for the Omicron period.

### Description of SARS-CoV-2-positive hospitalized patients

The median age (interquartile range [IQR]) was 53 (37–66) years for the pre-Omicron period and 33 (24–54) years for the Omicron period (Table 1). Most individuals had at least one risk factor for severe COVID-19 (1078 [80.3%] for the pre-Omicron period, 315 [71.6%] for the Omicron period). The majority of individuals received oxygen therapy (1299 [72.9%]), and some received invasive mechanical ventilation (263 [14.8%]). Most individuals improved and discharged (1074 [80.0%] for the pre-Omicron period and 320 [72.7%] for the Omicron period) (Table 1). However, in-hospital death occurred in 252 (18.8%) for the pre-Omicron period and 114 (25.9%) for the Omicron period.

**Table 1.** Demographic and clinical characteristics of hospitalized COVID-19 cases and factors associated with in-hospital death during the pre-Omicron (Alpha, Gamma, and Delta) and Omicron periods in San Lazaro Hospital, Philippines

	All (*n* = 1782)	Pre-Omicron (*n* = 1342)	Omicron (*n* = 440)	Adjusted odds ratios for in-hospital death (95% CI)^a^
Median age in years^b^	49 (32–64)	53 (37–66)	33(24–54)	N/A
Age in years, *n* (%)				
0–9	81 (4.6)	30 (2.2)	51 (11.6)	0.31 (0.10–0.97)
10–19	74 (4.2)	37 (2.8)	37 (8.4)	0.92 (0.38–2.26)
20–29	194 (10.9)	109 (8.1)	85 (19.3)	1
30–39	311 (17.5)	212 (15.8)	99 (22.5)	1.53 (0.87–2.71)
40–49	247 (13.9)	201 (15.0)	46 (10.5)	2.03 (1.11–3.71)
50–59	296 (16.6)	252 (18.8)	44 (10.0)	2.01 (1.10–3.65)
60–69	308 (17.3)	273 (20.3)	35 (8.0)	2.94 (1.63–5.32)
70–79	179 (10.0)	155 (11.6)	24 (5.5)	4.54 (2.43–8.46)
80–89	82 (4.6)	67 (5.0)	15 (3.4)	4.96 (2.43–10.14)
≥90	10 (0.6)	6 (0.5)	4 (0.9)	5.16 (0.92–28.9)
Sex, *n* (%)				
Female	698 (39.2)	556 (41.4)	142 (32.3)	1
Male	1084 (60.8)	786 (58.6)	298 (67.7)	1.60 (1.17–2.17)
Pregnancy at hospitalization among females, *n* (%)				
No	677 (97.0)	545 (98.0)	132 (93.0)	1
Yes	21 (3.0)	11 (2.0)	10 (7.0)	Could not be estimated
Healthcare worker, *n* (%)				
No	1673 (93.9)	1250 (93.1)	423 (96.1)	1
Yes	109 (6.1)	92 (6.9)	17 (3.9)	0.25 (0.07–0.85)
Comorbidities, *n* (%)^c^				
Cardiovascular disease	759 (42.6)	671 (50.0)	88 (20.0)	0.83 (0.60–1.17)
Diabetes mellitus	371 (20.8)	325 (24.2)	46 (10.5)	1.18 (0.82–1.68)
Dyslipidaemia	56 (3.1)	49 (3.7)	7 (1.6)	0.88 (0.38–2.03)
Chronic kidney disease	20 (1.1)	14 (1.0)	6 (1.4)	4.39 (1.52–12.67)
Chronic liver disease	4 (0.2)	2 (0.2)	2 (0.5)	3.44 (0.43–27.8)
Chronic obstructive pulmonary disease	15 (0.8)	9 (0.7)	6 (1.4)	0.55 (0.13–2.29)
Cancer	20 (1.1)	13 (1.0)	7 (1.6)	2.36 (0.79–7.06)
Dementia	7 (0.4)	5 (0.4)	2 (0.5)	Could not be estimated
Depression/schizophrenia	3 (0.2)	3 (0.2)	0 (0.0)	6.64 (0.37–120.0)
Stroke	44 (2.5)	32 (2.4)	12 (2.7)	1.30 (0.62–2.74)
Down syndrome	2 (0.1)	0 (0.0)	2 (0.5)	Could not be estimated
Tuberculosis	306 (17.2)	128 (9.5)	178 (40.5)	2.45 (1.69–3.57)
HIV infection	162 (9.1)	43 (3.2)	119 (27.1)	3.30 (2.03–5.37)
Immunodeficiency without HIV	1 (0.1)	0 (0.0)	1 (0.2)	Could not be estimated
Immunosuppressant use	2 (0.1)	0 (0.0)	2 (0.5)	4.47 (0.24–84.0)
Body mass index in kg/m^2^, *n* (%) (among individuals over 18 years of age with data available)				
<25	819 (55.8)	560 (48.4)	259 (83.3)	1
25–29 (overweight)	393 (26.8)	359 (31.0)	34 (10.9)	0.90 (0.61–1.32)
≥30 (obese)	257 (17.5)	230 (20.6)	18 (5.8)	0.72 (0.44–1.18)
Hospitalization in the past year, *n* (%)				
No	1680 (94.3)	1290 (96.1)	390 (88.6)	1
Yes	102 (5.7)	52 (3.9)	50 (11.4)	3.38 (2.01–5.67)
Past SARS-CoV–2 infection, *n* (%)				
None	1746 (97.9)	1330 (99.1)	416 (94.6)	1
Once	35 (2.0)	11 (0.8)	24 (5.5)	0.40 (0.11–1.52)
Twice	1 (0.1)	1 (0.1)	0 (0.0)	Could not be estimated
Smoking, *n* (%)				
Never-smoker	995 (55.8)	828 (61.7)	167 (38.0)	1
Past smoker	177 (9.9)	131 (9.8)	46 (10.5)	1.56 (0.98–2.48)
Current smoker	205 (11.5)	126 (9.4)	79 (18.0)	2.65 (1.72–4.10)
Underage	166 (9.3)	73 (5.4)	93 (21.1)	N/A
Unknown	239 (13.4)	184 (13.7)	55 (12.5)	N/A
Vaccination status, *n* (%); missing 310 (17.4%)				
Unvaccinated	865 (58.8)	687 (66.2)	178 (41.0)	Refer to VE evaluation later
Partially vaccinated	147 (10.0)	124 (12.0)	23 (5.3)	Refer to VE evaluation later
Primary series	410 (27.9)	226 (21.8)	184 (42.4)	Refer to VE evaluation later
First booster	44 (3.0)	1 (0.1)	43 (9.9)	Refer to VE evaluation later
Second booster	6 (0.4)	0 (0.0)	6 (1.4)	Refer to VE evaluation later
Symptoms, *n* (%)				
Fever above 37.5°C	1230 (69.0)	985 (73.4)	245 (55.7)	N/A
Malaise	626 (35.1)	515 (38.4)	111 (25.2)	N/A
Chills	82 (4.6)	57 (4.3)	25 (5.7)	N/A
Joint and body aches	228 (12.8)	178 (13.3)	50 (11.4)	N/A
Headache	243 (13.6)	191 (14.2)	52 (11.8)	N/A
Runny nose	347 (19.5)	287 (21.4)	60 (13.6)	N/A
Cough	1339 (75.4)	1061 (79.1)	278 (63.2)	N/A
Sore throat	224 (12.6)	194 (14.5)	30 (6.8)	N/A
Shortness of breath	934 (52.4)	742 (55.3)	192 (43.6)	N/A
Vomiting, diarrhoea, stomachache	355 (19.9)	235 (17.5)	120 (27.3)	N/A
Loss of taste or smell	172 (9.7)	170 (12.7)	2 (0.5)	N/A
Oxygen or invasive mechanical ventilation use, *n* (%)				
No oxygen	483 (27.1)	330 (24.6)	153 (34.8)	N/A
Oxygen only	1036 (58.1)	838 (62.4)	198 (45.0)	N/A
Invasive mechanical ventilation use	263 (14.8)	174 (13.0)	89 (20.2)	N/A
Outcome, *n* (%)				
Improved and discharged	1394 (78.2)	1074 (80.0)	320 (72.7)	N/A
Improved and transferred	3 (0.2)	1 (0.1)	2 (0.5)	N/A
Stable and transferred	6 (0.3)	6 (0.5)	0 (0.0)	N/A
Worsened and transferred	2 (0.1)	1 (0.1)	1 (0.2)	N/A
In-hospital death	366 (20.5)	252 (18.8)	114 (25.9)	N/A
Discharge against medical advice	11 (0.6)	8 (0.6)	3 (0.7)	N/A
Hospitalization length (days)^b^	10 (6–14)	10 (7–14)	9 (4–15)	N/A
Oxygen use length (days)^b^	6 (3–10)	7 (3–11)	4 (1–9)	N/A
Ventilation use length (days)^b^	1 (2–7)	2 (1–8)	2 (1–5)	N/A

a Adjusted for age group, sex, risk score category (0, 1, 2, 3–4, 5+), calendar week of hospitalization (biweekly), and vaccine doses (except for the factor of interest); estimated only for baseline characteristics before infection.

b Median (interquartile range).

c Odds ratio compared to not having each condition as a reference

Abbreviations: CI, confidence interval; N/A, not applicable.

### Factors associated with in-hospital death among SARS-CoV-2-positive hospitalized patients

Among hospitalized cases, older age was associated with in-hospital death in an incremental manner (compared to individuals who were in their 20s; adjusted odds ratio [aOR] for 40s: 2.03 [95% confidence interval {CI} 1.11–3.71]; aOR for 50s: 2.01 [95% CI 1.10–3.65]; aOR for 60s: 2.94 [95% CI 1.10–3.65]; aOR for 70s: 4.54 [95% CI 2.43–8.46]; aOR for 80s: 4.96 [95% CI 2.43–10.1]; aOR for <10 years of age: 0.31 [95% CI 0.10–0.97]; *p*-value for trend: *P* < 0.001) (Table 1). Other factors associated with in-hospital death included male sex (aOR 1.60 [95% CI 1.17–2.17]); the comorbidities of chronic kidney disease (aOR 4.39 [95% CI 1.52–12.67]), tuberculosis (aOR 2.45 [95% CI 1.69–3.57]), and HIV infection (aOR 3.30 [95% CI 2.03–5.37]); hospitalization in the past year (aOR 3.38 [95% CI 2.01–5.67]); and current smoker (aOR 2.65 [95% CI 1.72–4.10]) (Table 1).

### Baseline characteristics for the vaccine effectiveness analysis

The median age (interquartile range [IQR]) was 64 (57–71) years for the pre-Omicron period and 64 (57–72) for the Omicron period, and it was similar between cases and controls (Table 2). Most individuals had at least one risk factor for severe COVID-19 (716 [88.9%] for the pre-Omicron period, 228 [89.8%] for the Omicron period). During the pre-Omicron period, 118 (56.7%) received CoronaVac (SinoVac), 43 (20.7%) received AZD1222 (AstraZeneca), 24 (11.5%) received Ad26.COV2.S (Janssen/J&J), 10 (4.8%) received BNT162b2 (Pfizer), 7 (3.4%) received mRNA-1273 (Moderna), and 2 (1.0%) received Sputnik V (Gameleya), with 4 (1.9%) unknown (Table 2). During the Omicron period, for the primary series, 72 (49.3%) received CoronaVac (SinoVac), 23 (15.3%) received AZD1222 (AstraZeneca), 18 (12.3%) received BNT162b2 (Pfizer), 18 (12.3%) received Ad26.COV2.S (Janssen/J&J), 10 (6.9%) received mRNA-1273 (Moderna), 1 (0.7%) received Sputnik V (Gameleya), and 1 (0.7%) received BBIBP-CorV (Sinopharm), with 3 (2.1%) unknown (Table 2). For the first booster, 14 (48.3%) received BNT162b2 (Pfizer), 6 (20.7%) received AZD1222 (AstraZeneca), and 5 (17.2%) received mRNA-1273 (Moderna), with 4 (13.8%) unknown. For the second booster, 3 (75.0%) received BNT162b2 (Pfizer), and 1 (25.0%) received mRNA-1273 (Moderna) (none were unknown).

**Table 2.** Demographic and clinical characteristics of individuals included in the vaccine effectiveness estimates during the pre-Omicron (Alpha, Gamma, and Delta) period and the Omicron period in San Lazaro Hospital, Philippines

	All (*n* = 805)	Test-positive (*n* = 750)	Test-negative (*n* = 55)
Pre-Omicron (Alpha, Gamma, and Delta) period			
Median age in years^a^	64 (57–71)	64 (57–71)	66 (58–74)
Age in years, *n* (%)			
50–59	266 (33.0)	250 (33.3)	16 (29.1)
60–69	288 (35.8)	272 (36.3)	16 (29.1)
70–79	173 (21.5)	155 (20.7)	18 (32.7)
80–89	72 (8.9)	67 (8.9)	5 (9.1)
≥90	6 (0.8)	6 (0.8)	0 (0.0)
Sex, *n* (%)			
Male	432 (53.7)	402 (53.6)	30 (54.6)
Female	373 (46.3)	348 (46.4)	25 (45.5)
Pregnancy at hospitalization, *n* (%)			
No	804 (99.9)	749 (99.9)	55 (100.0)
Yes	1 (0.1)	1 (0.1)	0 (0.0)
Comorbidities, *n* (%)			
Cardiovascular disease	549 (68.2)	510 (68.0)	39 (70.9)
Diabetes mellitus	280 (34.8)	259 (34.5)	21 (38.2)
Dyslipidaemia	38 (4.7)	38 (5.1)	0 (0.0)
Chronic kidney disease	13 (1.6)	10 (1.3)	3 (5.5)
Chronic liver disease	2 (0.3)	2 (0.3)	0 (0.0)
Chronic obstructive pulmonary disease	10 (1.2)	9 (1.2)	1 (1.8)
Cancer	10 (1.2)	9 (1.2)	1 (1.8)
Dementia	6 (0.8)	5 (0.7)	1 (1.8)
Depression/schizophrenia	2 (0.3)	2 (0.3)	0 (0.0)
Stroke	32 (4.0)	27 (3.6)	5 (9.1)
Down syndrome	0 (0.0)	0 (0.0)	0 (0.0)
Tuberculosis	73 (9.1)	60 (8.0)	13 (2.7)
HIV infection	8 (1.0)	7 (0.9)	1 (1.8)
Immunodeficiency without HIV	0 (0.0)	0 (0.0)	0 (0.0)
Immunosuppressant use	0 (0.0)	0 (0.0)	0 (0.0)
Body mass index in kg/m^2^, *n* (%)			
<25	376 (52.4)	348 (52.0)	28 (57.1)
25–29 (overweight)	218 (30.4)	208 (31.1)	10 (20.4)
≥30 (obese)	124 (17.3)	113 (16.9)	11 (22.5)
Severe disease risk score^b^, *n* (%)			
0	89 (11.1)	84 (11.2)	5 (9.1)
1	225 (28.0)	212 (28.3)	13 (23.6)
2	152 (18.9)	143 (19.1)	9 (16.4)
3	166 (20.6)	154 (20.5)	12 (21.8)
≥4	173 (21.5)	157 (20.9)	16 (29.1)
Hospitalization in the past year, *n* (%)			
No	778 (96.7)	730 (97.3)	48 (87.3)
Yes	27 (3.4)	20 (2.7)	7 (12.7)
Smoking, *n* (%)			
Never-smoker	514 (63.9)	482 (64.3)	32 (58.2)
Past smoker	102 (12.7)	93 (12.4)	9 (16.4)
Current smoker	81 (10.7)	70 (9.3)	11 (20.0)
Unknown	108 (13.4)	105 (14.0)	3 (5.5)
Number of COVID–19 vaccinations received,^c^ *n* (%); missing 204 (25.3%)			
Unvaccinated	393 (65.4)	361 (64.5)	32 (78.1)
Partially vaccinated	77 (12.8)	75 (13.4)	2 (4.9)
Primary series	131 (21.8)	124 (22.1)	7 (17.1)
First booster	0 (0.0)	0 (0.0)	0 (0.0)
Vaccine type (primary series), *n* (%)^d^			
CoronaVac (SinoVac)	118 (56.7)	113 (56.8)	5 (55.6)
AZD1222 (AstraZeneca)	43 (20.7)	40 (20.1)	3 (33.3)
Ad26.COV2.S (Janssen/J&J)	24 (11.5)	23 (11.6)	1 (11.1)
BNT162b2 (Pfizer)	10 (4.8)	10 (5.0)	0 (0.0)
mRNA–1273 (Moderna)	7 (3.4)	7 (3.5)	0 (0.0)
Sputnik V (Gameleya)	2 (1.0)	2 (1.0)	0 (0.0)
Unknown	4 (1.9)	4 (2.0)	0 (0.0)
SARS-CoV–2 testing type, *n* (%)			
Nucleic acid amplification test	782 (97.1)	729 (97.2)	53 (96.4)
Rapid antigen detection kit	20 (2.5)	18 (2.4)	2 (3.6)
Unknown	3 (0.4)	3 (0.4)	0 (0.0)
Omicron period			

	All (*n* = 254)	Test positive (*n* = 119)	Test negative (*n* = 135)
Median age in years^a^	64 (57–72)	64 (57–73)	63 (57–71)
Age in years, *n* (%)			
50–59	94 (37.0)	43 (36.1)	51 (37.8)
60–69	79 (31.1)	35 (29.4)	44 (32.6)
70–79	45 (17.7)	23 (19.3)	22 (16.3)
80–89	29 (11.4)	14 (11.8)	15 (11.1)
≥90	7 (2.8)	4 (3.4)	3 (2.2)
Sex, *n* (%)			
Male	161 (63.4)	74 (62.2)	87 (64.4)
Female	93 (36.6)	45 (37.8)	48 (35.6)
Pregnancy at hospitalization, *n* (%)			
No	254 (100.0)	119 (100.0)	135 (100.0)
Yes	0 (0.0)	0 (0.0)	0 (0.0)
Comorbidities, *n* (%)			
Cardiovascular disease	118 (46.5)	64 (53.8)	54 (40.0)
Diabetes mellitus	64 (25.2)	35 (29.4)	29 (21.5)
Dyslipidaemia	4 (1.6)	4 (3.4)	0 (0.0)
Chronic kidney disease	8 (3.2)	4 (3.4)	4 (3.0)
Chronic liver disease	0 (0.0)	0 (0.0)	0 (0.0)
Chronic obstructive pulmonary disease	16 (6.3)	5 (4.2)	11 (8.2)
Cancer	8 (3.2)	5 (4.2)	3 (2.2)
Dementia	3 (1.2)	2 (1.7)	1 (0.7)
Depression/schizophrenia	0 (0.0)	0 (0.0)	0 (0.0)
Stroke	16 (6.3)	10 (8.3)	6 (4.4)
Down syndrome	1 (0.4)	0 (0.0)	1 (0.7)
Tuberculosis	125 (49.2)	46 (38.7)	79 (58.5)
HIV infection	8 (3.2)	3 (2.5)	5 (3.7)
Immunodeficiency without HIV	0 (0.0)	0 (0.0)	0 (0.0)
Immunosuppressant use	0 (0.0)	0 (0.0)	0 (0.0)
Body mass index in kg/m^2^, *n* (%)			
<25	178 (79.8)	78 (76.5)	100 (82.6)
25–29 (overweight)	28 (12.6)	17 (16.7)	11 (9.1)
≥30 (obese)	17 (7.6)	7 (6.9)	10 (8.3)
Severe disease risk score^b^, *n* (%)			
0	26 (10.2)	16 (13.5)	10 (7.4)
1	86 (33.9)	30 (25.2)	56 (41.5)
2	61 (24.0)	28 (23.5)	33 (24.4)
3	41 (16.1)	26 (21.9)	15 (11.1)
≥4	40 (15.8)	19 (16.0)	21 (15.6)
Hospitalization in the past year, *n* (%)			
No	237 (93.3)	111 (93.3)	126 (93.3)
Yes	17 (6.7)	8 (6.7)	9 (6.7)
Smoking, *n* (%)			
Never smoker	109 (42.9)	55 (46.2)	54 (40.0)
Past smoker	42 (16.5)	23 (19.3)	19 (14.1)
Current smoker	72 (28.4)	25 (21.0)	47 (34.8)
Unknown	31 (12.2)	16 (13.5)	15 (11.1)
Number of COVID–19 vaccinations received, *n* (%); missing 4 (1.6%)			
Unvaccinated	104 (41.6)	54 (45.8)	50 (37.9)
Partially vaccinated	7 (2.8)	4 (3.4)	3 (2.3)
Primary series	110 (44.0)	45 (38.1)	65 (49.2)
First booster	25 (10.0)	13 (11.0)	12 (9.1)
Second booster	4 (1.6)	2 (1.7)	2 (1.5)
Vaccine type (primary series), *n* (%)			
CoronaVac (SinoVac)	72 (49.3)	35 (54.7)	37 (45.1)
AZD1222 (AstraZeneca)	23 (15.8)	9 (14.1)	14 (17.1)
BNT162b2 (Pfizer)	18 (12.3)	7 (10.9)	11 (13.4)
Ad26.COV2.S (Janssen/J&J)	18 (12.3)	7 (10.9)	11 (13.4)
mRNA–1273 (Moderna)	10 (6.9)	4 (6.3)	6 (7.3)
Sputnik V (Gameleya)	1 (0.7)	1 (1.6)	0 (0.0)
BBIBP-CorV (Sinopharm)	1 (0.7)	0 (0.0)	1 (1.2)
Unknown	3 (2.1)	1 (1.6)	2 (2.4)
Vaccine type (first booster), *n* (%)			
BNT162b2 (Pfizer)	14 (48.3)	9 (60.0)	5 (35.7)
AZD1222 (AstraZeneca)	6 (20.7)	3 (20.0)	3 (21.4)
mRNA–1273 (Moderna)	5 (17.2)	2 (13.3)	3 (21.4)
Unknown	4 (13.8)	1 (6.7)	3 (21.4)
Vaccine type (second booster), *n* (%)			
BNT162b2 (Pfizer)	3 (75.0)	2 (100.0)	1 (50.0)
mRNA–1273 (Moderna)	1 (25.0)	0 (0.0)	1 (50.0)
SARS-CoV–2 testing type, *n* (%)			
Nucleic acid amplification test	241 (94.9)	111 (93.3)	130 (96.3)
Rapid antigen detection kit	9 (3.5)	5 (4.2)	4 (3.0)
Unknown	4 (1.6)	3 (2.5)	1 (0.7)

a Median (interquartile range).

b The following points were added up for each patient: assigned 2 points for the presence of either diabetes mellitus, chronic kidney disease, dementia, Down syndrome, or obesity and assigned 1 point for the presence of cardiovascular disease (including hypertension), dyslipidaemia, chronic liver disease, chronic obstructive pulmonary disease, cancer, depression/schizophrenia, stroke, pregnancy while hospitalized, or overweight.

### Vaccine effectiveness against all COVID-19 hospitalization, COVID-19 requiring oxygen therapy, COVID-19 requiring mechanical ventilation, and fatal COVID-19

During the pre-Omicron period, VE estimates for 2 doses were 85.4% (95% CI 35.9–96.7%) against all COVID-19 hospitalizations, 91.0% (95% CI 49.4–98.4%) against COVID-19 requiring oxygen therapy, 97.0% (95% CI 65.7–99.7%) against COVID-19 requiring invasive mechanical ventilation, and 96.5% (95% CI 67.1–99.6%) against fatal COVID-19 (Table 3). During the Omicron period, VE estimates for 2 doses were 70.2% (95% CI 27.0–87.8%) against all COVID-19 hospitalization, 71.4% (95% CI 29.3–88.4%) against COVID-19 requiring oxygen therapy, 72.7% (95% CI −11.6–93.3%) against COVID-19 requiring invasive mechanical ventilation, and 58.9% (95% CI −82.8–90.8%) against fatal COVID-19 (Table 3). During the Omicron period, some individuals received 3 or 4 doses, but the confidence intervals were very wide due to the small sample size. Similarly, we attempted to estimate VE by time since vaccination, but failed to estimate some, and even if we could, the confidence intervals were wide (by dose in Supplementary Table 1, regardless of dose in Supplementary Table 2).

**Table 3.** Vaccine effectiveness against various COVID-19 hospitalization outcomes by the number of doses received during the pre-Omicron (Alpha, Gamma, and Delta) and Omicron periods in San Lazaro Hospital, Philippines

Vaccination status	Cases, *n*	Controls, *n*	Median time since vaccination, days^a^	Adjusted odds ratios (95% CI)^b^	Vaccine effectiveness, % (95% CI)^c^
Pre-Omicron: all COVID–19 hospitalization					
Unvaccinated	361	32	N/A	1	N/A
Partially vaccinated	75	2	19 (12–31)	1.800 (0.356–9.098)	N/A
Primary series	124	7	65 (34–108)	0.146 (0.033–0.641)	85.4 (35.9–96.7)
Pre-Omicron: COVID–19 requiring oxygen therapy					
Unvaccinated	318	32	N/A	1	N/A
Partially vaccinated	57	2	20 (13–30)	1.430 (0.260–7.873)	N/A
Primary series	95	7	64 (38–104)	0.090 (0.016–0.506)	91.0 (49.4–98.4)
Pre-Omicron: COVID–19 requiring oxygen therapy, restricted to patients with respiratory failure due to COVID–19					
Unvaccinated	314	32	N/A	1	N/A
Partially vaccinated	57	2	20 (13–30)	1.440 (0.261–7.929)	N/A
Primary series	95	7	64 (38–104)	0.091 (0.016–0.511)	90.9 (48.9–98.4)
Pre-Omicron: COVID–19 requiring invasive mechanical ventilation					
Unvaccinated	80	32	N/A	1	N/A
Partially vaccinated	6	2	19 (11–31)	0.188 (0.140–2.541)	N/A
Primary series	13	7	59 (36–110)	0.030 (0.003–0.343)	97.0 (65.7–99.7)
Pre-Omicron: fatal COVID–19					
Unvaccinated	114	32	N/A	1	N/A
Partially vaccinated	7	2	14 (11–30)	0.707 (0.073–6.821)	N/A
Primary series	19	7	60 (28–106)	0.035 (0.004–0.329)	96.5 (67.1–99.6)
Omicron: all COVID–19 hospitalization					
Unvaccinated	54	50	N/A	1	N/A
Partially vaccinated	4	3	76 (36–213)	0.930 (0.101–8.592)	N/A
Primary series	45	65	172 (142–294)	0.298 (0.122–0.730)	70.2 (27.0–87.8)
First booster	13	12	84 (28–281)	1.402 (0.337–5.837)	N/A
Second booster	2	2	Could not be estimated		
Omicron: COVID–19 requiring oxygen therapy					
Unvaccinated	53	50	N/A	1	N/A
Partially vaccinated	3	3	102 (50–213)	0.661 (0.062–7.063)	N/A
Primary series	31	65	177 (148–359)	0.286 (0.116–0.707)	71.4 (29.3–88.4)
First booster	5	12	197 (75–321)	0.752 (0.155–3.650)	N/A
Second booster	0	2	Could not be estimated		
Omicron: COVID–19 requiring oxygen therapy, restricted to patients with respiratory failure due to COVID–19					
Unvaccinated	51	50	N/A	1	N/A
Partially vaccinated	3	3	102 (50–213)	0.636 (0.058–6.945)	N/A
Primary series	29	65	182 (149–362)	0.231 (0.090–0.595)	76.9 (40.5–91.0)
First booster	5	12	197 (75–321)	0.690 (0.140–3.388)	N/A
Second booster	0	2	Could not be estimated		
Omicron: COVID–19 requiring invasive mechanical ventilation					
Unvaccinated	19	50	N/A	1	N/A
Partially vaccinated	2	3	158 (76–227)	9.725 (0.232–408.111)	N/A
Primary series	7	65	269 (149–473)	0.273 (0.067–1.116)	72.7 (−11.6–93.3)
First booster	1	12	93 (75–197)	0.427 (0.028–6.631)	N/A
Second booster	0	2	Could not be estimated		
Omicron: fatal COVID–19					
Unvaccinated	20	50	N/A	Could not be estimated	
Partially vaccinated	1	3	213 (50–240)	Could not be estimated	
Primary series	11	65	265 (153–456)	0.411 (0.092–1.828)	58.9 (−82.8–90.8)
First booster	1	12	93 (75–197)	0.126 (0.009–1.868)	87.4 (−86.8–99.1)
Second booster	0	2	Could not be estimated		

a Median (interquartile range); among individuals with available vaccination dates.

b Adjusted for age group, sex, risk score category (0, 1, 2, 3–4, 5+), smoking history, and calendar week of hospitalization (biweekly).

c Effectiveness estimates are provided when the confidence intervals are ±100%.

Abbreviations: CI, confidence interval; N/A, not applicable.

## Discussion

In this descriptive and case-control study in the Philippines, we described the characteristics and outcomes of COVID-19 patients requiring hospitalization and estimated the real-world effectiveness of COVID-19 vaccines against severe disease during the pre-Omicron and Omicron periods.

Among SARS-CoV-2-positive hospitalized patients, in-hospital death occurred in 20.5%, which was in line with what was observed in a systematic review/meta-analysis published early in the pandemic [23], although cautious interpretation is warranted given varied hospitalization criteria among countries and hospitals. The numerically higher percentage of in-hospital deaths during the Omicron period (25.9%) compared to the pre-Omicron period (18.8%) may be partially due to numerically higher percentages of individuals with either TB (pre-Omicron: 9.5% versus Omicron: 40.5%) or HIV (pre-Omicron: 3.2% versus Omicron: 27.1%). We found several factors associated with in-hospital death, including increasing age, male sex (aOR 1.60), CKD (aOR 4.39), tuberculosis (aOR 2.45), HIV (aOR 3.30), hospitalization in the past year (aOR 3.38), and current smokers (aOR 2.65). All these are in line with previous reports [21, 22, 24–26], although these findings were new in LMICs in the Western Pacific Region and Southeast Asia.

Next, in the VE analysis, during the pre-Omicron period, over half (56.7%) of vaccinees received CoronaVac, 32.2% received viral vector vaccines, and 8.2% received mRNA vaccines (Table 2). With these vaccine types, 2 doses provided high (85–97%) protection for a range of severe COVID-19 outcomes during the pre-Omicron (Alpha, Gamma, and Delta) period for the approximate median interval since the last vaccination of 2 months (all hospitalization: 85.4%; oxygen requirement: 91.0% [restricted to ‘true’ severe COVID-19: 90.9%]; invasive mechanical ventilation: 97.0%; fatal: 96.5%) (Table 3). These findings were in agreement with other observational studies [13], including studies that assessed inactivated vaccines such as CoronaVac [14]. Also, a trend towards higher VE for more severe and specific outcomes was observed [15, 16].

During the Omicron period, approximately half (49.3%) of the primary series vaccinees received CoronaVac, 27.6% received viral vector vaccines, and 19.2% received mRNA vaccines (Table 2). For boosters, the majority received either mRNA or viral vector vaccines (only mRNA vaccines for the second booster doses). Here, 2 doses also provided variable moderate-to-high (59–77%) protection (all hospitalization: 70.2%; oxygen requirement: 71.4% [restricted to ‘true’ severe COVID-19: 76.9%]; invasive mechanical ventilation: 72.7%; fatal: 58.9% [some with wide CI]) (Table 2). The numerically lower VE against more severe outcomes such as mechanical ventilation and death may be due to a longer period since the last vaccination (median interval of approximately 9 months versus 6 months) in addition to small sample sizes. Unfortunately, we could not estimate VE for booster doses, VE by vaccine type (e.g. manufacturers), and VE by time since vaccination in detail, due to sample size limitations.

The strengths of the current study include analyzing data from an understudied country, data on different vaccine platforms, and outcome data across different severity levels.

### Limitations

This study has several limitations. First, biases, confounding, and misclassifications inherent in observational studies are possible. However, using specific and severe outcomes, we aimed to minimize the inclusion of incidental SARS-CoV-2-positive cases which could have occurred as admission screening was in place at the time of the study. Second, the current hospital-based case-control study was not strictly a test-negative design, as controls included all patients who required oxygen even for severe outcomes such as mechanical ventilation use and death. However, individuals who require oxygen therapy are likely to seek care regardless of SARS-CoV-2 infection or vaccination status due to shortness of breath and other manifestations, resulting in the same advantage of control for healthcare-seeking behaviour. Third, the present study was a single-centre study, and thus, the results may not be generalizable to the whole country. Fourth, wide CIs for some estimates warrant careful interpretation of point estimates, and the small sample size in some multivariable models resulted in possible sparse data bias. Fifth, our analysis was a complete case analysis with more missing data during the pre-Omicron period, as the first version of the CIF for SARS-CoV-2 testing used during this period did not include vaccination information. However, it is possible that these patients with missing data were unvaccinated (being early in the course of the vaccination rollout), and we obtained very similar VE estimates for various outcomes when we treated missing as unvaccinated (data not shown). Also, this missing proportion is comparable to data-linkage studies [27]. Sixth, we could not classify individual COVID-19 cases as infected with specific variants during the pre-Omicron period. Seventh, our VE estimates measured within a median of 2 months during the pre-Omicron period and 6–9 months during the Omicron period. Finally, as above, we could not estimate VE by vaccine type (e.g. manufacturer) due to sample size limitations, but we consider this is still of value to see the context in the Philippines.

## Conclusions

In this descriptive and case-control study in the Philippines, we identified increasing age, male sex, certain comorbidities (CKD, tuberculosis, and HIV), hospitalization in the past year, and current smoking as factors associated with in-hospital death among hospitalized COVID-19 patients. Also, VE estimates against severe COVID-19 resulting in hospitalization, oxygen, mechanical ventilation, and death were high for 6 months during both the pre-Omicron and Omicron periods in a setting where over half of vaccinees received inactivated vaccines for the primary series. Our findings will support policies implemented in lower-middle and low-income countries, where many rolled out inactivated vaccines but with scarce real-world data.

## Supporting information

Arashiro et al. supplementary material Arashiro et al. supplementary material

## Data Availability

Individual-level data of patients included in this manuscript after de-identification are considered sensitive and will not be shared. The study methods and statistical analyses are all described in detail in the Methods and throughout the manuscript.

## Supplementary material

For supplementary material accompanying this paper visit http://doi.org/10.1017/S0950268824001845. click here to view supplementary material
