# Supplementary material for: Factors associated with COVID-19 in-hospital death and COVID-19 vaccine effectiveness against COVID-19 hospitalization in the Philippines during pre-omicron and omicron period: A case-control study (MOTIVATE-P study)
Source: Epidemiol Infect. 2024 Dec 20;153:e18. doi: 10.1017/S0950268824001845 (PMC11748018; doi:10.1017/S0950268824001845)
Supplement: Arashiro et al. supplementary material [file S0950268824001845sup001.docx]

**Supplementary Table 1.** Vaccine effectiveness against various COVID-19 hospitalization outcomes by dose and time since vaccination during the pre-Omicron (Alpha, Gamma, Delta) and Omicron periods in San Lazaro Hospital, Philippines

| Vaccination status | Cases,  n | Controls,  n | Median time since vaccination, days^a^ | Adjusted odds ratios (95% CI)^b^ | Vaccine effectiveness, % (95% CI)^c^ |
| --- | --- | --- | --- | --- | --- |
| Pre-Omicron: all COVID-19 hospitalization | | | | | |
| Unvaccinated | 361 | 32 | N/A | 1 | N/A |
| Within 13 days of partial vaccination | 24 | 0 | Could not be estimated | | |
| 14 days after partial vaccination or within 13 days of primary series | 57 | 3 | 25 (16–38) | 0.511 (0.112–2.318) | N/A |
| 14 days to 6 months after primary series | 103 | 4 | 73 (40–110) | 0.222 (0.043–1.150) | 77.8 (-15.0–95.7) |
| >6 months after primary series | 1 | 1 | 191 (190–192) | 0.014 (0.0002–1.030) | 98.6 (-3.0–99.98) |
| Pre-Omicron: COVID-19 requiring oxygen therapy | | | | | |
| Unvaccinated | 318 | 32 | N/A | 1 | N/A |
| Within 13 days of partial vaccination | 16 | 0 | Could not be estimated | | |
| 14 days after partial vaccination or within 13 days of primary series | 44 | 3 | 24 (15–34) | 0.423 (0.086–2.077) | N/A |
| 14 days to 6 months after primary series | 81 | 4 | 67 (41–104) | 0.207 (0.040–1.083) | 79.3 (-8.3–96) |
| >6 months after primary series | 1 | 1 | 191 (190–192) | 0.015 (0.0002–1.042) | 98.5 (-4.2–99.98) |
| Pre-Omicron: COVID-19 requiring invasive mechanical ventilation | | | | | |
| Unvaccinated | 80 | 32 | N/A | 1 | N/A |
| Within 13 days of partial vaccination | 3 | 0 | Could not be estimated | | |
| 14 days after partial vaccination or within 13 days of primary series | 5 | 3 | 19 (11–31) | 0.024 (0.001–0.427) | 97.6 (57.3–99.9) |
| 14 days to 6 months after primary series | 9 | 4 | 67 (49–110) | 0.077 (0.005–1.107) | 92.3 (-10.7–99.5) |
| >6 months after primary series | 0 | 1 | Could not be estimated | | |
| Pre-Omicron: fatal COVID-19 | | | | | |
| Unvaccinated | 114 | 32 | N/A | 1 | N/A |
| Within 13 days of partial vaccination | 4 | 0 | Could not be estimated | | |
| 14 days after partial vaccination or within 13 days of primary series | 6 | 3 | 14 (10–30) | 0.071 (0.006–0.774) | 92.9 (22.6–99.4) |
| 14 days to 6 months after primary series | 15 | 4 | 67 (50–106) | 0.066 (0.006–0.758) | 93.4 (24.2–99.4) |
| >6 months after primary series | 0 | 1 | Could not be estimated | | |
| Omicron: all COVID-19 hospitalization | | | | | |
| Unvaccinated | 54 | 50 | N/A | 1 | N/A |
| Partial or within 13 days of primary series | 4 | 2 | 76 (36–213) | 1.852 (0.325–10.555) | N/A |
| 14 days to 6 months after primary series | 19 | 9 | 144 (116–163) | 1.955 (0.810–4.720) | N/A |
| >6 months after primary series | 10 | 16 | 319 (240–475) | 0.579 (0.240–1.393) | 42.1 (-39.3–76) |
| Within 13 days of first booster | 1 | 0 | Could not be estimated | | |
| 14 days to 6 months after first booster | 2 | 2 | 46 (26–78) | 0.926 (0.126–6.824) | N/A |
| >6 months after first booster | 3 | 1 | 301 (239–321) | 2.778 (0.280–27.585) | N/A |
| Within 13 days of second booster | 0 | 0 | Could not be estimated | | |
| 14 days to 6 months after second booster | 1 | 1 | 53 (31–75) | 0.926 (0.056–15.202) | N/A |
| >6 months after second booster | 0 | 0 | Could not be estimated | | |
| Omicron: COVID-19 requiring oxygen therapy | | | | | |
| Unvaccinated | 53 | 50 | N/A | 1 | N/A |
| Partial or within 13 days of primary series | 3 | 2 | 102 (50–213) | 1.100 (0.080–15.142) | N/A |
| 14 days to 6 months after primary series | 13 | 9 | 148 (136–164) | 0.642 (0.111–3.711) | N/A |
| >6 months after primary series | 6 | 16 | 359 (265–487) | 0.273 (0.044–1.688) | 72.7 (-68.8–95.6) |
| Within 13 days of first booster | 0 | 0 | Could not be estimated | | |
| 14 days to 6 months after first booster | 0 | 2 | Could not be estimated | | |
| >6 months after first booster | 3 | 1 | Could not be estimated | | |
| Within 13 days of second booster | 0 | 0 | Could not be estimated | | |
| 14 days to 6 months after second booster | 0 | 1 | Could not be estimated | | |
| >6 months after second booster | 0 | 0 | Could not be estimated | | |
| Omicron: COVID-19 requiring invasive mechanical ventilation | | | | | |
| Unvaccinated | 19 | 50 | N/A | 1 | N/A |
| Partial or within 13 days of primary series | 2 | 2 | 158 (76–227) | 20.994 (0.356-1237.956) | N/A |
| 14 days to 6 months after primary series | 3 | 9 | 144 (129–158) | 0.881 (0.071–10.875) | N/A |
| >6 months after primary series | 2 | 16 | 421 (294–516) | 1.131 (0.060–21.283) | N/A |
| Within 13 days of first booster | 0 | 0 | Could not be estimated | | |
| 14 days to 6 months after first booster | 0 | 2 | Could not be estimated | | |
| >6 months after first booster | 1 | 1 | Could not be estimated | | |
| Within 13 days of second booster | 0 | 0 | Could not be estimated | | |
| 14 days to 6 months after second booster | 0 | 1 | Could not be estimated | | |
| >6 months after second booster | 0 | 0 | Could not be estimated | | |
| Omicron: fatal COVID-19 | | | | | |
| Unvaccinated | 20 | 50 | N/A | 1 | N/A |
| Partial or within 13 days of primary series | 1 | 2 | Could not be estimated | | |
| 14 days to 6 months after primary series | 5 | 9 | 148 (141–164) | 193.205 (0.822–45422.870) | N/A |
| >6 months after primary series | 3 | 16 | 385 (270–516) | 0.814 (0.011–62.734) | N/A |
| Within 13 days of first booster | 0 | 0 | Could not be estimated | | |
| 14 days to 6 months after first booster | 0 | 2 | Could not be estimated | | |
| >6 months after first booster | 1 | 1 | Could not be estimated | | |
| Within 13 days of second booster | 0 | 0 | Could not be estimated | | |
| 14 days to 6 months after second booster | 0 | 1 | Could not be estimated | | |
| >6 months after second booster | 0 | 0 | Could not be estimated | | |

^a^ Median (interquartile range); among individuals with available vaccination dates

^b^ Adjusted for age group, sex, risk score category (0, 1, 2, 3-4, 5+), smoking history, and calendar week of hospitalization (biweekly).

^c^ Effectiveness estimates are provided when the confidence intervals are ±100%

Abbreviations: CI, confidence interval; N/A, not applicable.

**Supplementary Table 2.** Vaccine effectiveness against various COVID-19 hospitalization outcomes by time since vaccination regardless of doses received during the pre-Omicron (Alpha, Gamma, Delta) and Omicron periods in San Lazaro Hospital, Philippines

| Vaccination status | Cases,  n | Controls,  n | Median time since vaccination, days^a^ | Adjusted odds ratios (95% CI)^b^ | Vaccine effectiveness, % (95% CI)^c^ |
| --- | --- | --- | --- | --- | --- |
| Pre-Omicron: all COVID-19 hospitalization | | | | | |
| Unvaccinated | 361 | 32 | N/A | 1 | N/A |
| Partially vaccinated | 75 | 2 | 19 (12–31) | 1.569 (0.303–8.133) | N/A |
| Within 13 days of vaccination | 9 | 1 | 9 (6–10) | 0.522 (0.037–0.733) | 47.8 (26.7–96.3) |
| 14 days to 6 months after vaccination | 103 | 4 | 73 (40–110) | 0.206 (0.038–1.112) | 79.4 (-11.2–96.2) |
| >6 months after vaccination | 1 | 1 | 191 (190–192) | 0.134 (0.0002–1.049) | 86.6 (-4.9–99.98) |
| Pre-Omicron: COVID-19 requiring oxygen therapy | | | | | |
| Unvaccinated | 318 | 32 | N/A | 1 | N/A |
| Partially vaccinated | 57 | 2 | 20 (13–30) | 1.280 (0.229–7.163) | N/A |
| Within 13 days of vaccination | 6 | 1 | 10 (8–10) | 0.022 (0.001–0.418) | 97.8 (58.2–99.9) |
| 14 days to 6 months after vaccination | 81 | 4 | 67 (41–104) | 0.166 (0.278–1.005) | 83.4 (-0.5–72.2) |
| >6 months after vaccination | 1 | 1 | 191 (190–192) | 0.013 (0.0001–1.018) | 98.7 (-1.8–99.99) |
| Pre-Omicron: COVID-19 requiring invasive mechanical ventilation | | | | | |
| Unvaccinated | 80 | 32 | N/A | 1 | N/A |
| Partially vaccinated | 6 | 2 | 19 (11–31) | 0.254 (0.020–3.211) | N/A |
| Within 13 days of vaccination | 2 | 1 | Could not be estimated | | |
| 14 days to 6 months after vaccination | 9 | 4 | 67 (49–110) | 0.040 (0.002–0.840) | 96.0 (16.0–99.8) |
| >6 months after vaccination | 0 | 1 | Could not be estimated | | |
| Pre-Omicron: fatal COVID-19 | | | | | |
| Unvaccinated | 114 | 32 | N/A | 1 | N/A |
| Partially vaccinated | 7 | 2 | 14 (11–30) | 0.653 (0.067–6.402) | N/A |
| Within 13 days of vaccination | 3 | 1 | 10 (9–11) | 0.032 (0.001–1.157) | 96.3 (-15.7–99.9) |
| 14 days to 6 months after vaccination | 15 | 4 | 67 (50–106) | 0.068 (0.006–0.770) | 93.2 (23–99.4) |
| >6 months after vaccination | 0 | 1 | Could not be estimated | | |
| Omicron: all COVID-19 hospitalization | | | | | |
| Unvaccinated | 54 | 50 | N/A | 1 | N/A |
| Partially vaccinated | 4 | 3 | 76 (36–213) | 1.013 (0.106–9.655) | N/A |
| Within 13 days of vaccination | 1 | 0 | Could not be estimated | | |
| 14 days to 6 months after vaccination | 22 | 12 | 138 (83–153) | 0.640 (0.154–2.663) | N/A |
| >6 months after vaccination | 13 | 17 | 308 (240–473) | 0.615 (0.137–2.759) | N/A |
| Omicron: COVID-19 requiring oxygen therapy | | | | | |
| Unvaccinated | 53 | 50 | N/A | 1 | N/A |
| Partially vaccinated | 3 | 3 | 102 (50–213) | 0.825 (0.072–9.457) | N/A |
| Within 13 days of vaccination | 0 | 0 | Could not be estimated | | |
| 14 days to 6 months after vaccination | 13 | 12 | 145 (121–164) | 0.368 (0.072–1.887) | 63.2 (-88.7–92.8) |
| >6 months after vaccination | 9 | 17 | 102 (50–213) | 0.648 (0.142–2.969) | N/A |
| Omicron: COVID-19 requiring invasive mechanical ventilation | | | | | |
| Unvaccinated | 19 | 50 | N/A | 1 | N/A |
| Partially vaccinated | 2 | 3 | 158 (76–227) | 34.030 (0.436–2655.073) | N/A |
| Within 13 days of vaccination | 0 | 0 | Could not be estimated | | |
| 14 days to 6 months after vaccination | 3 | 12 | 141 (93–153) | 0.505 (0.046–5.501) | N/A |
| >6 months after vaccination | 3 | 17 | 374 (276–502) | 2.886 (0.239–34.918) | N/A |
| Omicron: fatal COVID-19 | | | | | |
| Unvaccinated | 20 | 50 | N/A | 1 | N/A |
| Partially vaccinated | 1 | 3 | Could not be estimated | | |
| Within 13 days of vaccination | 0 | 0 | Could not be estimated | | |
| 14 days to 6 months after vaccination | 5 | 12 | 145 (121–162) | 5.151 (0.172–153.960) | N/A |
| >6 months after vaccination | 4 | 17 | 362 (270–487) | 12.469 (0.417–372.684) | N/A |

^a^ Median (interquartile range); among individuals with available vaccination dates

^b^ Adjusted for age group, sex, risk score category (0, 1, 2, 3-4, 5+), smoking history, and calendar week of hospitalization (biweekly).

^c^ Effectiveness estimates are provided when the confidence intervals are ±100%

Abbreviations: CI, confidence interval; N/A, not applicable.
